# Magnetic Resonance Imaging: A Tool for Pork Pie Development

**DOI:** 10.3390/foods2030393

**Published:** 2013-08-28

**Authors:** Adam P. Gaunt, Robert H. Morris, Michael I. Newton

**Affiliations:** School of Science & Technology, Nottingham Trent University, Nottingham NG11 8NS, UK; E-Mails: adam.gaunt2009@my.ntu.ac.uk (A.P.G.); michael.newton@ntu.ac.uk (M.I.N.)

**Keywords:** magnetic resonance imaging, MRI, pork pie, diffusion, jelly

## Abstract

The traditional British pork pie consists of roughly chopped pork cooked in a hot water pastry crust. Due to shrinkage of the meat during cooking, the gap formed around the meat is usually sealed using a gelatin based jelly to exclude air and thus help to preserve the pie. The properties of the jelly are such that it will ingress into the pastry crust causing undesirable softening. The jelly is traditionally produced by simmering pig trotters with seasoning for several hours. In this work we demonstrate the potential of magnetic resonance imaging (MRI) as a tool for investigating the conditions required for producing jellies with different properties and present two examples of this use. Firstly we demonstrate that MRI can determine the ability of water to diffuse through the jelly which is critical in minimizing the amount of moisture moving from the jelly to the crust. Secondly, the impact of jelly temperature on the penetration length into the crust is investigated. These examples highlight the power of MRI as a tool for food assessment.

## 1. Introduction

Magnetic Resonance Imaging (MRI), most well-known for medical applications, has been increasing in popularity as a technique for use in the assessment of food and drink products [1,2,3,4,5,6,7,8,9,10,11,12,13,14,15]. The ability to show the location of fat and water and determine if it is free or bound is a significant help in the analysis of such complex systems. The measurement of the transverse (T_2_) and longitudinal (T_1_) relaxation times and the ability to measure the proton self-diffusion coefficient (D_0_) provide a powerful tool to understand a number of structural and organoleptic properties of foodstuffs such as textural properties, component stability, viscosity and water distribution. Examples of food studies using MRI include the nature of water distribution in bread during storage [1], the characteristics of cooked and cured meats [2,3,4,5,6,7,8,9,10,11,12], the characterization of water mobility and distribution in cereals and cookies [13], quality control and sensory quantification of dairy products [14,15] and recent review articles [16,17]. Work such as these examples is important to the industries that fund the research in order to improve the shelf life of products and to optimize their production. To the author’s knowledge, there have been no previous papers that have examined the effect of hot jelly on a hot water pastry crust such as that found in the pork pie. The traditional British pork pie consists of roughly chopped pork cooked in a hot water pastry crust, with one particular variant having been recognized by the European Union protection of geographical indications and designations of origin for agricultural products and foodstuffs such as the Melton Mowbray pork pie [18]. Due to shrinkage of the meat during cooking, the gap formed around the meat is usually sealed using a jelly to exclude air and help preserve the pie and maintain favorable organoleptic properties by maintaining desired moisture content. This same moisture stabilization which is desirable in the meat component has a negative effect on the pastry crust which softens over time. The jelly is traditionally produced by simmering pig trotters and bones with seasoning for several hours. A previous study investigated in detail the properties of such pies [19] but did not use MRI in their assessment. In this work we demonstrate that MRI provides a powerful tool for determining the ideal conditions for minimizing the amount of moisture moving from the jelly to the crust, resulting in an undesirable softening of the pie crust.

## 2. Experimental Section

The jelly was produced by boiling 2 kg of coarsely chopped pig trotters in 6.5 L of water for 2 h. After this time the pig’s trotters were removed and the solution was allowed to boil for a further 2 h during which the jelly was reduced as a result of the water boiling off. We refer to this period as the concentration time, during which samples were taken every 15 min. There were 5 samples of 10 mL taken at each of 9 concentration times which were sieved to filter particulate matter before being stored in 10 mL glass screw top vials. Once the samples were collected they were stored in a refrigerator at 6 ± 2 °C in order to prevent the gelatin structure from breaking down. The viscosities of the jellies were determined using a CSL2 rheometer (Brookfield Engineering, MA, USA). The pastry samples were made from a traditional hot water paste: 500 g of plain flour, 2.5 g of salt, 300 g of lard and 300 mL of water. To combine the ingredients the lard and the water were heated together until all the lard was melted before slowly adding it to the flour and salt. The mixture was then mixed until malleable and cool enough to handle. Dough samples were baked in a 5 cm diameter and 2 cm deep cupcake mould to give them a similar shape to a traditional pork pie without the meat filling. A circular cutter was used to get an even sample size of 6.5 cm in diameter. Once samples were cut out and placed in the mould, a small weight was placed inside the pastry to mimic the effect of the meat, ensuring that the sample kept its shape and to prevent the pastry rising. The pastry was placed in an oven at 220 °C for 30 min, after this time the anti-rising weight was removed and the samples were left to bake for a further 10 min. This replicates the conditions experienced by the area of the pastry which is most susceptible to moisture changes, the side walls of the pastry.

Once the samples were cooked to the point where the pastry appeared golden brown they were removed from the oven, and the mould, and left to cool. The jelly was removed from the heat and filtered before cooling after which 5 mL of one of 9 different concentrations was added to the pastry with samples produced in triplicate. The temperature was monitored (using a thermal imaging camera (i5, FLIR, OR, USA) and a thermocouple probe (1602, Comark Instruments, UK)) as it cooled to allow jelly samples to be taken from 70 to 30 °C in steps of 10 °C. The jelly was immediately added to the pastry samples and left to cool before scanning. This method was carried out for each concentration.

All MR Images were collected using a small bore Bruker Biospec MRI scanner with a magnetic field of 2.35T. Three different scan protocols were used in the experiments. The two main imaging scans were Single Point Imaging (SPI) [20] and Rapid Acquisition with Relaxation Enhancement (RARE) [21], which were used to obtain images of the pastry samples combined with the jelly. Both of these sequences generate spin echoes to produce an image with T_2_ or T_2_^eff^ contrast respectively. SPI is a slow sequence (11 min per image for this study) in which each point in k-space requires an excitation pulse in contrast to RARE which collects a number of k-space lines for a single excitation pulse making it very rapid (16 s in this study). SPI is used because it allows for collection of very short T_2_ values (on the order of milliseconds) to which RARE is not sensitive (the shortest measurable value is on the order of 50 ms. The third imaging protocol was a diffusion tensor imaging sequence [22] allowing diffusion maps to be calculated; this was used to find the diffusion coefficients of the jelly samples. The images were thresholded by signal intensity to show the area within the pastry that contained jelly. These images were compared to control images to determine the penetration length into the crust.

## 3. Results and Discussion

**Figure 1:**
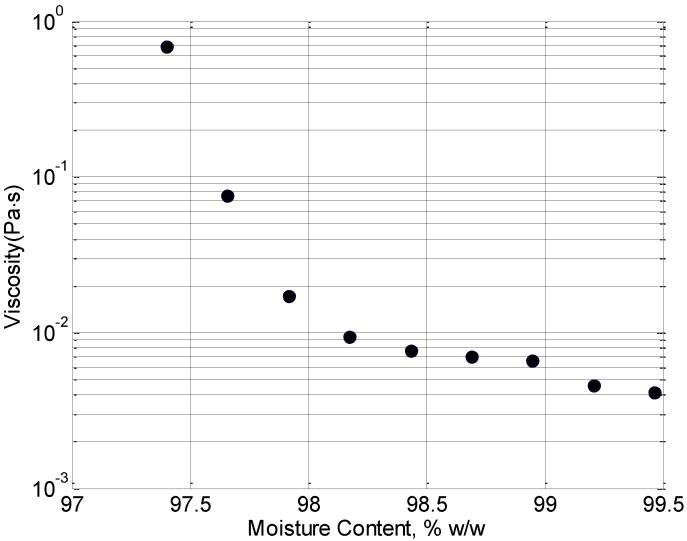
Viscosity of the jelly as a function of moisture content.

**Figure 2:**
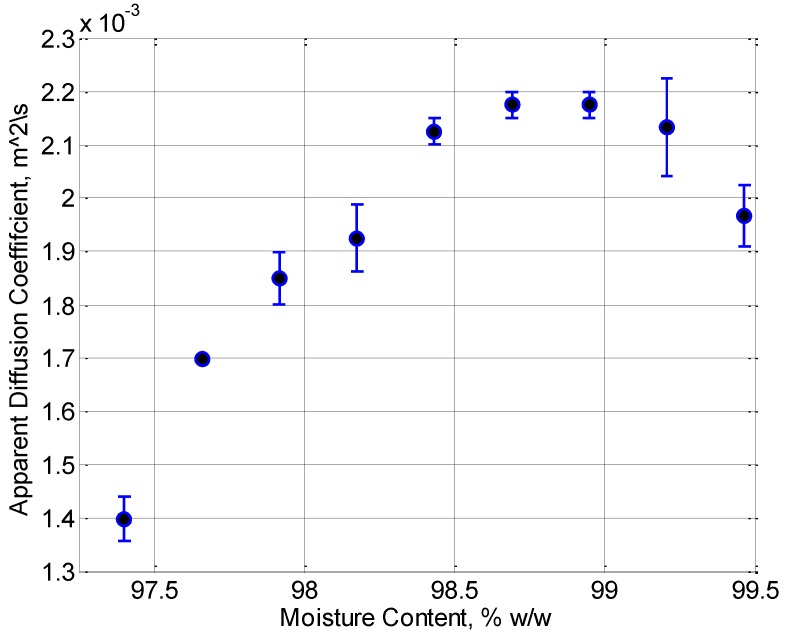
MRI measured proton diffusion coefficient as a function of moisture content.

The viscosity of the jelly as a function of concentration time is shown in Figure 1. As expected, the longer the jelly is simmered, the more the water content is reduced and hence the more viscous it becomes. The trotters used for jelly preparation are a natural product and as such are subject to variation however repeats with different batches show consistent trends. Figure 2 shows how the diffusion of the water molecules become more limited as concentration time is increased due to the increased viscosity and the increased binding of gelatin molecules in the water. It is most likely that the initial increase in the apparent diffusion coefficient (ADC) around the 99% point is due to an error in the fitting procedure. The ADC is estimated by fitting the experimental data points, collected at different pulsed field gradient intensities, to a model of free diffusion. At the 99% moisture content, it is probable that this model does not hold completely, resulting in a spurious result. The overall trend in increasing viscosity and decreasing diffusion are both expected to lead to a reduction in the penetration length.

**Figure 3:**
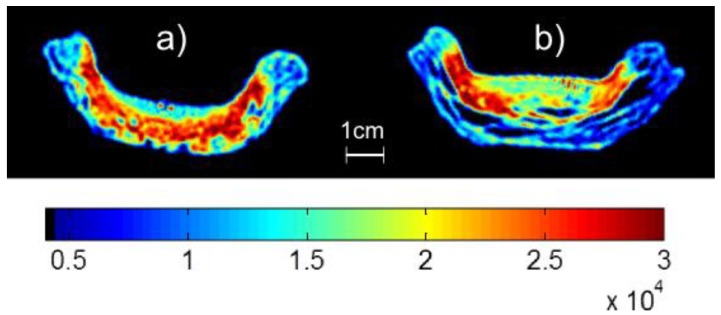
Single Point Images of pastry with jelly produced after concentration times of (**a**) 30 min and (**b**) 120 min corresponding to moisture contents of 98.9% and 97.4% respectively. The color scale represents the MR signal intensity with blue representing low signal intensity (e.g., pastry without jelly due to low moisture content) through green to red which represents high signal intensity (e.g., jelly due to high moisture content).

Two example MRI images of pie crusts which have had samples of jelly at 70 °C introduced to their surface are shown in Figure 3 for concentration times of 30 and 120 min. This temperature was chosen as it represents the greatest penetration as it is the highest temperature explored in this work. Red areas highlight where there are areas of mobile moisture in the pastry and yellow areas suggest bound water. Note how the longer concentration time results in a visible jelly layer above the pastry.

**Figure 4:**
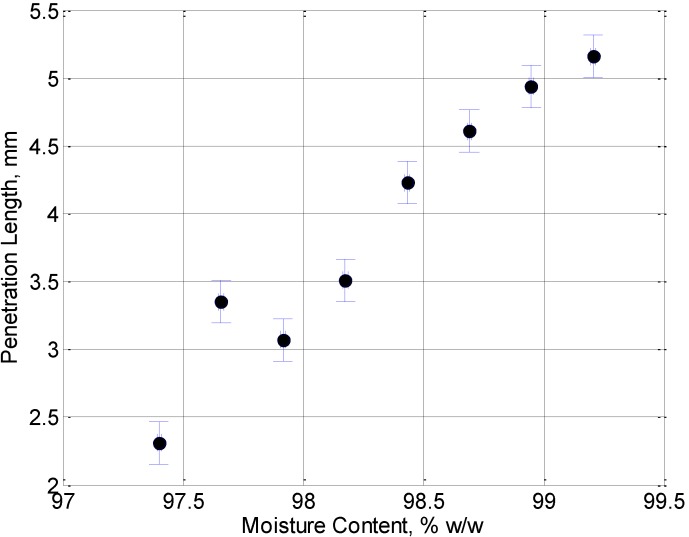
The penetration length of jelly into the pie crust at a temperature of 70 °C plotted as a function of the moisture content.

At this temperature, the jelly sample that has been concentrated for 30 min almost entirely enters into the crust. For increased concentration times some of the jelly remains on the surface. Figure 4 shows a plot of this penetration length into the surface of the pastry crusts as a function of moisture content. The point at a moisture content of 97.65% sits higher than the trend formed by the other points. This is most likely due to insufficient mixing of the jelly before collecting the samples at this concentration time. It can however be clearly seen that the lower the moisture content, the less the jelly will move into the crust. Data presented here is for one set of jellies produced in the same batch.

**Figure 5:**
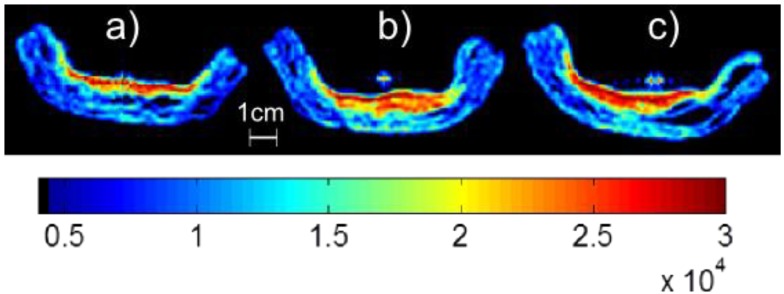
Single Point Images of pastry and jelly samples, where the jelly has a concentration time of 90 min. The temperatures of the jellies at the time of introducing to the crusts were (**a**) 30 °C, (**b**) 50 °C, and (**c**) 70 °C. The color scale represents the MR signal intensity with blue representing low signal intensity (e.g., pastry without jelly due to low moisture content) through green to red which represents high signal intensity (e.g., jelly due to high moisture content).

**Figure 6:**
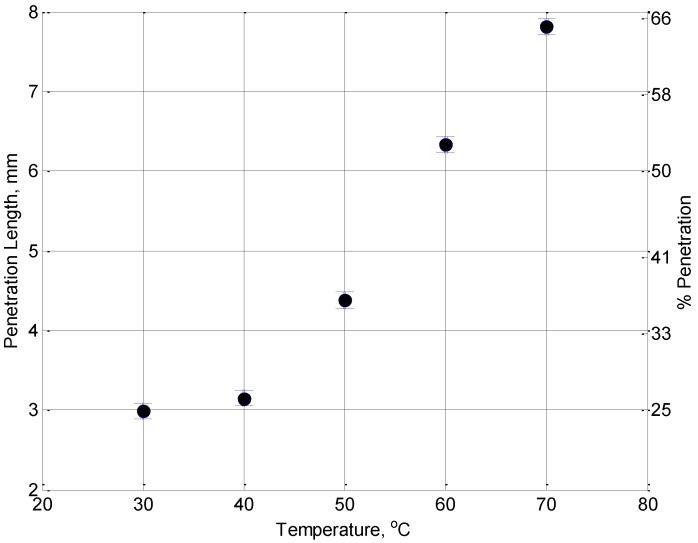
The penetration length of jelly into the pie crust with a concentration time of 90 minutes plotted as a function of the jelly temperature. The right hand axis shows this as a percentage of the total thickness of the pastry.

Figure 5 shows single point images collected for a jelly with a concentration time of 90 min applied at a range of temperatures. At the lowest temperature, the jelly remains closer to the surface than for the higher temperatures. The length that the jelly has penetrated into the crust as a function of the temperature for the remaining samples is shown in Figure 6. It can be seen that the lower the temperature of the jelly, the less it penetrates into the sample, although for temperatures lower than the setting point of the jelly this will not be the case. For real applications, there is a lower threshold for this temperature set at 45 °C to prevent microbial contamination of the product [19].

## 4. Conclusions

In this work we have shown that MRI may be used to determine the extent to which water diffuses within traditionally produced jelly. This is an important factor in minimizing the amount of moisture moving from the jelly to the crust of a pork pie, resulting in an undesirable pie crust softening. In our focus on demonstrating the suitability of MRI measurements to this application we have not included the taste or mouth feel which would be required to provide all of the information for product development. Investigating the effect of the temperature of the jelly added to the crust has shown that the penetration length of the jelly into the pie crust can be extracted from the MRI images and that lowering the temperature that the jelly is added at, could provide a possible means of reducing penetration. Although images have not been included here, we have also shown the ability of MRI to determine if the jelly has completely sealed in the meat or if there is still air contact. This could be a useful tool for online process monitoring of pie production. We have demonstrated in this work that MRI is indeed a powerful tool for product development in the food processing industry and has the potential to provide useful information that could lead to product improvements. 
